# Feasibility and Safety of Pressurized Intraperitoneal Aerosol Chemotherapy for Peritoneal Carcinomatosis: A Retrospective Cohort Study

**DOI:** 10.1155/2017/6852749

**Published:** 2017-02-26

**Authors:** Martin Hübner, Hugo Teixeira Farinha, Fabian Grass, Anita Wolfer, Patrice Mathevet, Dieter Hahnloser, Nicolas Demartines

**Affiliations:** ^1^Department of Visceral Surgery, University Hospital of Lausanne (CHUV), Lausanne, Switzerland; ^2^Department of Medical Oncology, University Hospital of Lausanne (CHUV), Lausanne, Switzerland; ^3^Department of Gynecology, University Hospital of Lausanne (CHUV), Lausanne, Switzerland

## Abstract

*Background*. Pressurized intraperitoneal aerosol chemotherapy (PIPAC) has been introduced as a novel repeatable treatment for peritoneal carcinomatosis. The available evidence from the pioneer center suggests good tolerance and high response rates, but independent confirmation is needed. A single-center cohort was analyzed one year after implementation for feasibility and safety. *Methods*. PIPAC was started in January 2015, and every patient was entered into a prospective database. This retrospective analysis included all consecutive patients operated until April 2016 with emphasis on surgical feasibility and early postoperative outcomes. *Results*. Forty-two patients (M : F = 8 : 34, median age 66 (59–73) years) with 91 PIPAC procedures in total (4×: 1,  3×: 17,  2×: 12, and  1×: 12) were analyzed. Abdominal accessibility rate was 95% (42/44); laparoscopic access was not feasible in 2 patients with previous HIPEC. Median initial peritoneal carcinomatosis index (PCI) was 10 (IQR 5–17). Median operation time was 94 min (89–108) with no learning curve observed. One PIPAC application was postponed due to intraoperative intestinal lesion. Overall morbidity was 9% with 7 minor complications (Clavien I-II) and one PIPAC-unrelated postoperative mortality. Median postoperative hospital stay was 3 days (2-3). *Conclusion*. Repetitive PIPAC is feasible in most patients with refractory carcinomatosis of various origins. Intraoperative complications and postoperative morbidity rates were low. This encourages prospective studies assessing oncological efficacy.

## 1. Introduction

Peritoneal carcinomatosis (PC) remains a condition with limited treatment options and dismal prognosis [1–3]. Outcome appears to be worse for PC compared to other stage IV situations, and response rates to systemic chemotherapy are modest at best, mainly due to limited tissue concentrations [4, 5]. Furthermore, side effects are common, and the use of palliative chemotherapy has therefore been questioned recently [6–8]. Hyperthermic intraperitoneal chemotherapy has been suggested in conjunction with cytoreductive surgery as an alternative in selected patients with encouraging results. However, most patients with PC are not eligible for this major procedure associated with important morbi-mortality [9, 10]. Furthermore, tissue concentrations after HIPEC remain low due to unequal distribution and low penetration [11].

Pressurized intraperitoneal aerosol chemotherapy (PIPAC) has been introduced as a novel treatment for peritoneal carcinomatosis [12, 13]. Pressure application allows for equal distribution and deeper penetration resulting in higher tissue concentrations despite lower doses (low systemic uptake) [14–16]. Minimally invasive access without cytoreduction decreases morbidity and allows for repetitive application. PIPAC is a very new technique, and first human application took place in November 2011 only. So far, all clinical reports but one came from the pioneer center in Herne, Germany [17–20].

The aim of this study was to analyze and report a consecutive cohort of PIPAC patients in our tertiary center with regard to feasibility and safety of the procedure.

## 2. Methods

The PIPAC program was started at the Department of Visceral Surgery of the University Hospital of Lausanne, Switzerland (CHUV), in January 2015, and was endorsed by the medical direction. Eligibility criteria for PIPAC treatment were persistent or progressive isolated peritoneal disease under or after at least one line of systemic treatment. Cytoreductive surgery and HIPEC as potentially curative treatment option was always the preferred choice in the absence of contraindications. Exceptionally, patients with predominating symptomatic PC and very limited disease elsewhere were considered. All patients were seen in the outpatient setting by a surgeon together with an oncologist to discuss all available treatment options. All indications were confirmed at the multidisciplinary tumor board. Patients received detailed oral and written information about the nature and risk of this novel procedure, and all patients provided written consent prior to surgery. All patients were treated in a palliative setting since long-term outcomes after PIPAC treatment is not yet available. This important point was explicitly stated in the preoperative information and consenting session. According to Swiss legislation and our institutional directive, all patients were further asked for their consent for the utilization of their clinical data in anonymous form (general consent).

This retrospective analysis included all consecutive patients scheduled for PIPAC from the beginning of the program (January 2015) until April 2016. Excluded were only those patients refusing to sign the general consent form. The study was approved by the Institutional Review Board (number 2016-00274), conducted and reported in compliance with the STROBE criteria (http://strobe-statement.org/), and registered online (http://www.researchregistry.com; UIN: 1577).

### 2.1. PIPAC Procedure (Figure 1 [21])

Surgical technique and safety considerations have been described in detail by the Herne group, and our institutional protocol adhered strictly to these empirical standards [13, 16, 22]. Briefly, pneumoperitoneum was established by open placement of one 10 mm and one 5 mm balloon trocar, additional 5 mm trocars only if needed for technical difficulty. The peritoneal carcinomatosis index (PCI) was documented, and representative peritoneal nodules were biopsied. Intraperitoneal chemotherapy was applied by the use of a pressure injector (Accutron HP-D, Medtron®, Saarbrücken, Germany) and a specific nebulizer (MicroPump®, CapnoPen®, Reger, Villingendorf, Germany) at 37°C for 30 min and under standard laparoscopic pressure of 12 mmHg [23]. PIPAC was administered repetitively (3× at least) at an interval of about 6 weeks. In line with current protocols, patients with PC of colorectal origin received oxaliplatin (92 mg/m^2^), while a combination of cisplatin (7.5 mg/m^2^) and doxorubicin (1.5 mg/m^2^) was applied for the other malignancies [17–19].

### 2.2. Data Management and Outcome Measures

Demographic information and surgical details were prospectively entered for all patients in a computerized coded database designed specifically for quality control of the PIPAC cohort. Demographic data included age, gender, comorbidities, and nutritional status. American Association of Anesthesiologists (ASA) physical status and Eastern Cooperative Oncology Group (ECOG) performance status were documented as validated tools to describe general condition [24]. Nutritional risk was assessed by the use of the Nutritional Risk Score (NRS 2002) [25].

Surgical information contained operation time, intraoperative complications, need for concomitant adhesiolysis, accessibility of the abdomen, and number of trocars. Extent of peritoneal disease was documented by the use of the peritoneal cancer index (PCI) [26], and volume of ascites was measured. Surgical stress was assessed using the E-PASS (Estimation of Physiologic Ability and Surgical Stress) score [27].

Postoperative morbidity was assessed until 30 days after surgery by use of the Clavien classification, and length of postoperative hospital stay was measured including readmissions. Outpatient appointments were scheduled for all patients at 30 days after each procedure for clinical follow-up and quality control. Moreover, each patient received an emergency phone number and was advised to establish contact if a problem arises.

### 2.3. Predefined Clinical Questions

Several comparisons and statistical correlations were defined a priori. Univariate analysis was compared between patients with only one PIPAC versus patients with repeated applications. This was done to define conditions rendering repetitive administration difficult.

Gynecological malignancies differ from digestive cancers in many ways including previous surgical and systemic treatments and distribution patterns of peritoneal carcinomatosis. As this has a potential impact on surgical difficulty and approach, surgical aspects between gynecological and digestive patients were compared.

Lastly, increasing peritoneal tumor load might complicate surgery and reflect more advanced disease with consecutive longer hospital stay. In order to test these hypotheses, we correlated PCI to operation time and postoperative hospital stay, respectively.

### 2.4. Statistical Analysis

Continuous variables were presented as mean with standard deviation (SD) or median value with range or interquartile range (IQR) as appropriate depending on the normality of the distribution and compared using Student's *t*-test and Mann-Whitney *U* test. Categorical variables were given as frequencies with percentages and compared with chi-square test. Spearman's test was used to measure correlations between continuous variables. A *p* value of <0.05 was considered to be statistically significant in all tests. Data analyses were generated using SPSS v20 statistical software (Chicago, IL, USA); graphics were developed using GraphPad Prism 7 (GraphPad Software Inc., La Jolla, CA, USA).

## 3. Results

**Figure 2:**
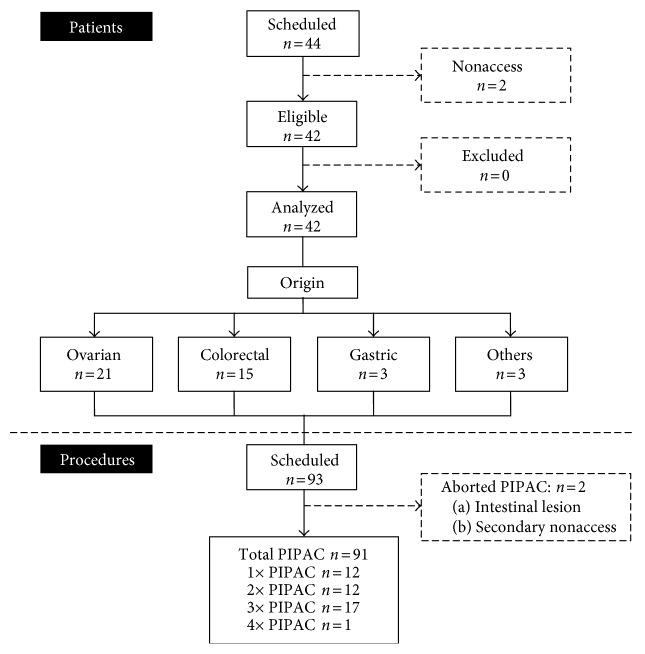
Flow of patients treated with pressurized intraperitoneal aerosol chemotherapy (PIPAC).

In the study period, 44 patients were scheduled for PIPAC. In 2 patients, no laparoscopic access could be established due to dense adhesions. All remaining patients had signed the general consent form, and no patient was excluded. Final analysis included therefore 42 patients (M : F = 8 : 34, median age 66 (IQR 59–73) years). Overall failure rate (number of nonaccess + aborted procedures/total number of attempted procedures) was 4/95 (4%) (Figure 2). Twenty-one patients (50%) had carcinomatosis of ovarian origin, 14 and 3 from colorectal and gastric cancer, respectively (remainder: 1 small bowel, 1 pseudomyxoma, and 1 mesothelioma). Demographic information is provided in Table 1.

Overall, 91 PIPAC procedures were performed; 18 patients had 3 or more PIPAC procedures, 12 patients had 2 operations, and 12 patients one procedure so far. Reasons for no 2nd PIPAC were progression of systemic disease in 5 patients, patient refusal in 3, absent peritoneal disease during first PIPAC in one, and secondary nonaccess during the 2nd PIPAC in one patient. The 2 remaining patients were awaiting their scheduled 2nd intervention. Patients with higher ASA score, lower BMI, underlying malnutrition, and colorectal origin were less likely to undergo repetitive PIPAC (Table 1).

### 3.1. Surgical Details (Table 2)

In 80 out of the 91 procedures (88%), one 5 mm and one 10 mm trocar were used; 3 trocars were needed in 11 surgeries. Median overall time for all procedures was 94 min (IQR 89–108) showing little variation over time. PIPAC procedures for gynecological peritoneal metastases were significantly shorter as compared with procedures for digestive PC. Surgical stress as measured by the E-PASS was a median of −0.20 (−0.32–−0.11). Median PCI was 10 (5–17), and adhesiolysis was necessary in 16% of cases before applying PIPAC. There was one intraoperative complication: a small bowel lesion occurred during open trocar placement. The enterotomy was recognized and repaired immediately. PIPAC was postponed and successfully performed six weeks later.

In patients with digestive origin, median PCI was significantly higher and operation time significantly longer.

### 3.2. Postoperative Outcomes

Eight complications occurred after 91 procedures, giving an overall morbidity rate of 8.8%. Seven minor complications were 3 urinary retentions with a need for 24 h catheterization, one ileus treated with nasogastric decompression, one minor scar bleeding, one constipation requiring enema, and one neutropenia with spontaneous resolution. One patient developed cardiogenic shock and arrhythmia 4 days after the 3rd PIPAC procedure with fatal outcome. Autopsy did not find any intra-abdominal complication, and no causative link could be established between PIPAC treatment and death.

Median hospital stay was 3 (IQR 2-3) days with 9 and 35 patients, respectively, who left the hospital on the 1st and 2nd postoperative days.

### 3.3. Correlation of Tumor Load, Operation Time, and Hospital Stay

**Figure 3:**
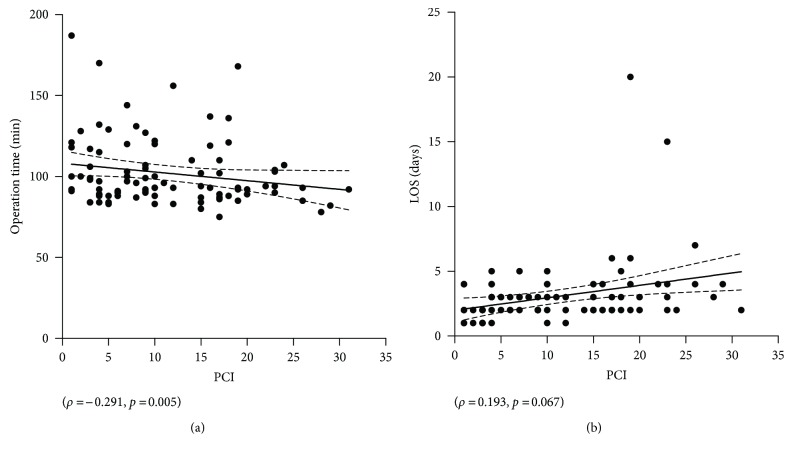
Correlation of tumor load with operation time and hospital stay. The extent of peritoneal disease (measured by the peritoneal cancer index: PCI) was plotted against operation time (a) and length of hospital stay (LOS) (b).

Higher PCI was significantly associated with shorter OR time (*ρ* = −0.291, *p* = 0.005), while no statistical correlation was found between PCI and hospital stay (*ρ* = 0.193, *p* = 0.067) (Figures 3(a) and 3(b)).

## 4. Discussion

In the present study, repeated PIPAC was feasible in most patients with peritoneal carcinomatosis. Postprocedure morbidity was low and hospital stay short.

Feasibility of PIPAC includes abdominal access by laparoscopy and repeated application. Minimally invasive surgery in patients with multiple prior surgeries is challenging and associated with higher conversion rates [28, 29]. For PIPAC in particular, the literature is very scarce. Reported primary nonaccess rates vary from 0 to 17%; after at least one PIPAC, secondary nonaccess rate was reported to be 0–35% [17–20]. In our series, we observed primary and secondary nonaccess rates of 4.5% and 2.4%, respectively; of note, both patients with primary nonaccess had prior HIPEC treatment. Repeatability is more complex and depends not only on technical problems but mostly on disease progression and preferences of patients and their care providers. Altogether, 10 out of 42 patients (23.8%) could not benefit from the 2nd PIPAC mostly due to the development of metastases other than peritoneal (*n* = 5) or for patient's wish to discontinue treatments (*n* = 3). Our findings are in line with the findings from the Herne group reporting repeated application in 64–82% of their patients [17–19].

Intraoperative complications appear to be exceedingly rare, but the risk for small bowel lesions is present in patients with adhesions as also reported by others [18]. Besides the mortality described in this paper, 2 fatal outcomes after PIPAC treatment have been reported in the literature [18]. Mortality was attributed to progressing small bowel disease causing obstruction in one patient and poor general condition with consecutive ascitic decompensation and renal failure in the other patient. Tempfer et al. suggested therefore that impeding small bowel obstruction and refractory ascites should be considered as contraindications to PIPAC treatment. Therefore, proper patient selection is important and challenging as patients tend to accept risks in view of lacking treatment alternatives in most cases. No CTCAE (Common Terminology Criteria for Adverse Events) grade 4 events were reported after PIPAC, while incidence of grade 3 events varied between 23 and 35% [17–20]. Applying the Clavien classification for postoperative complications, we observed an overall morbidity of 9% in our series. Postoperative abdominal pain, which was present in the Herne experience in up to 100% [17], required rarely other analgesics than those routinely used after other laparoscopic procedures like cholecystectomy. The median hospital stay was 3 days, similar as reported by an Italian group [20]. Reporting a considerable number of patients leaving hospital already 1-2 days after surgery, even outpatient surgery might be considered in the future for well-selected patients in good general condition. However, it must be underlined that the usual PIPAC patient is frail and in reduced general condition due to the disease and previous treatments. Caution is advocated especially for malnourished patients presenting with ascites as postoperative fluid shifts can be important entailing consecutive electrolyte disturbances and renal failure. But even those negatively selected patients required rarely more than 3 days after surgery before being fit for discharge.

Extent of peritoneal disease (as measured by the PCI) was in the present experience not associated with increased operation time, rather the contrary. Most patients with advanced peritoneal disease had considerable amounts of ascites facilitating abdominal access and also choice of appropriate location for biopsies. To our knowledge, impact of PCI on operation time has not been investigated by other groups. The present study did not observe any significant correlation between PCI and postoperative stay; these findings confirm that PIPAC treatment is feasible and safe within short hospital stay even for patients with very advanced disease [20].

Selection and reporting bias are important limitations of retrospective studies. The present analysis included all consecutive patients without any exclusion. All reported endpoints were defined a priori and documented online in a prospectively maintained database designed for quality control. The study sample however was modest and heterogeneous. Heterogeneity concerns especially prior surgical and systemic chemotherapy treatments which is a methodological problem in studies on peritoneal carcinomatosis in general. Comparisons with other cohorts treated intraperitoneally or systemically are hence problematic. Therefore, further data is certainly needed to confirm the present findings even if they are in line with data published so far.

In conclusion, the present study suggests that repetitive PIPAC is feasible in most patients with refractory carcinomatosis of various origins. Intraoperative events and postoperative complications are low. These findings encourage designing prospective studies assessing oncological efficacy.

## Figures and Tables

**Figure 1:**
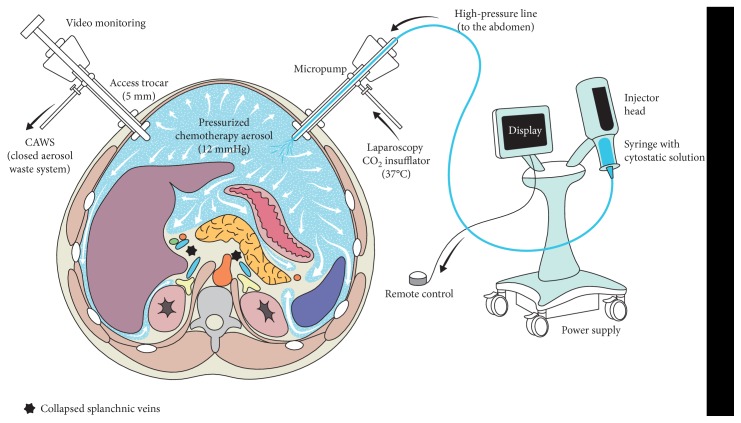
Pressurized intraperitoneal aerosol chemotherapy (PIPAC). The abdominal cavity is accessed with 2 balloon trocars allowing hermetic seal. Liquid chemotherapy is dispersed as aerosol by use of a standard injector and a specific nebulizer. Reprinted from *Rev Med Suisse* [21] with permission from Médicine et Hygiène.

**Table 1:** Baseline demographics of patients treated with pressurized intraperitoneal aerosol chemotherapy (PIPAC).

	All patients (*n* = 42)	1 PIPAC (*n* = 12)	2 or >3 PIPAC (*n* = 30)	*p* value
Demographics				
Median age (years)	66 (59–73)	62 (52–88)	67 (61–63)	0.185
Age ≥ 70 years	16 (38%)	4 (33%)	12 (40%)	0.687
Gender (male)	8 (19%)	4 (33%)	4 (13%)	0.135
Median BMI (kg/m^2^)	22.5 (20–25)	19 (19–23)	22.7 (21.3–26)	*0.018*
BMI < 18.5 kg/m^2^	2 (5%)	1	1	0.491
Comorbidities				
ASA (I-II)	28 (66%)	5 (42%)	23 (77%)	*0.029*
ECOG (0-1)	36 (86%)	9 (75%)	27 (90%)	0.209
Diabetes	1 (2%)	1	0	0.109
Malnutrition	14 (33%)	7 (58%)	7 (23%)	*0.029*
NRS < 3	29 (69%)	6 (50%)	23 (77%)	0.091
Previous laparotomy	1 (0–4)	1 (0–3)	1 (0–4)	*0.040*
≥2	15 (36%)	2 (16%)	13 (43%)	0.103
Disease				
Origin				*0.007*
Colorectal	15 (33%)	8 (66%)	6 (20%)	
Gastric	3 (7%)	0	3 (10%)	
Gynecological	21 (50%)	2 (17%)	19 (63%)	
Other	3	1	2	
Prior chemotherapy				0.229
No chemo	2	1	1	
1 line	9 (21%)	5 (42%)	4 (13%)	
2 lines	13 (31%)	2 (17%)	11 (36%)	
3 lines	9 (21%)	1	8 (26%)	
More than 3	9 (21%)	3 (25%)	6 (20%)	
Prior HIPEC	4 (10%)	2 (17%)	2 (7%)	0.318
Diagnosis—1st PIPAC (mo)	16 (1–104)	18 (1–73)	16 (1–104)	0.928

Median (range) for previous laparotomy and diagnosis—1st PIPAC, otherwise median (IQR) or number (%) as appropriate. Statistical significance (*p* < 0.05) is highlighted in italics.

BMI: body mass index; ASA: American Association of Anesthesiologists physical status classification system; ECOG: Eastern Cooperative Oncology Group performance status; HIPEC: hyperthermic intraperitoneal chemotherapy.

**Table 2:** Surgical details of pressurized intraperitoneal aerosol chemotherapy (PIPAC).

	Overall (*n* = 91)	GYN (*n* = 51)	Digestive (*n* = 40)	*p* value
Surgical feasibility				
Number of trocars	2 (2-3)	2 (2-3)	2 (2-3)	0.668
Operation time	94 (89–108)	91 (87–97)	100 (92–117)	*0.002*
Intra-OP findings				
PCI	10 (5–17)	9 (4–14)	15 (7–19)	*0.002*
Ascites (mL)	50 (0–4000)	0 (0–300)	50 (0–4000)	0.982
Adhesiolysis	15 (16%)	9 (18%)	6 (15%)	0.735
Median E-PASS	−0.20 (−0.32–−0.11)	−0.20 (−0.31–−0.10)	−0.20 (−0.31–−0.09)	0.733

Median (range) for number of trocars and ascites and median (IQR) for operation time, PCI, and E-PASS. Statistical significance (*p* < 0.05) is highlighted in italics.

PCI: peritoneal cancer index; E-PASS: Modified Estimation of Physiologic Ability and Surgical Stress.

## Abbreviations

IQR: Interquartile range
PC: Peritoneal carcinomatosis
PCI: Peritoneal cancer index
PIPAC: Pressurized intraperitoneal aerosol chemotherapy.
